# An In‐Silico Study to Identify Relevant Biomarkers in Sepsis Applying Integrated Bulk RNA Sequencing and Single‐Cell RNA Sequencing Analyses

**DOI:** 10.1002/gch2.202400321

**Published:** 2025-03-13

**Authors:** Qile Ye, Yuhang Dong, Jingting Liang, Jingyao Lv, Rong Tang, Shuai Zhao, Guiying Hou

**Affiliations:** ^1^ Department of Critical Care Medicine The Second Affiliated Hospital of Harbin Medical University Harbin 150001 China; ^2^ Department of Critical Care Medicine The Fourth Affiliated Hospital of Harbin Medical University Harbin 150001 China; ^3^ Department of Neurology Beidahuang Industry Group General Hospital Harbin 150088 China; ^4^ College of Basic Medicine Qiqihar Medical University Qiqihar 161006 China; ^5^ Intensive Care Unit Ruikang Hospital Affiliated to Guangxi University of Chinese Medicine Nanning 530011 China; ^6^ Department of Respiratory and Critical Care Medicine The Second Affiliated Hospital of Harbin Medical University Harbin 150001 China

**Keywords:** bulk RNA sequencing analysis, cell–cell communication, monocytes, sepsis, single‐cell RNA sequencing analysis

## Abstract

This study aims to discover sepsis‐related biomarkers via in‐silico analyses. The single‐cell sequencing RNA (sc‐RNA) data and metabolism‐related genes are obtained from public databases and previous studies, respectively. Cell subpopulations are identified and annotated, followed by performing single‐sample geneset enrichment analysis (ssGSEA and identification of differentially expressed genes (DEGs). Weighted gene co‐expression network analysis (WGCNA) is applied to classify specific gene modules, and the key module is subjected to immune infiltration analysis. The communication between the subclusters of monocytes is visualized. Five cell subpopulations (subcluster C1‐5) containing a relatively higher percentage of monocytes are identified, with subcluster C4 having the lowest enrichment score of metabolism‐related genes. Genes with a higher expression in the subclusters are enriched for antigen processing and presentation of exogenous antigen, lymphocyte differentiation, and leukocyte activation. Subcluster C5 affected other subclusters through galectin 9 (LGALS9)‐CD45 and LGALS9‐CD44, while other subclusters affected subcluster C5 through MIF‐(CD74+C‐X‐C motif chemokine receptor 4 (CXCR4)) and MIF‐(CD74+CD44). Six genes (F‐Box Protein 4, *FBXO4*; Forkhead Box K1, *FOXK1*; MSH2 with MutS Homolog 2, *MSH2*; Nop‐7‐associated 2, *NSA2*; Transmembrane Protein 128, *TMEM128*; and *SBDS*) are determined as the hub genes for sepsis. The 6 hub genes are positively correlated with, among others, monocytes and NK cells, but negatively correlated with neutrophils. This study identifies accurate biomarkers for sepsis, contributing to the diagnosis and treatment of the disease.

## Graphical abstract

Integrated bulk RNA sequencing and single‐cell RNA sequencing contributes to the identification of metabolism‐related biomarkers in sepsis.

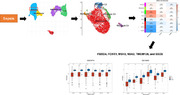

## 1 Introduction

Sepsis is a life‐threatening condition characterized by organ dysfunction caused by an uncontrolled host response to infection. The diagnosis of sepsis is particularly challenging due to the presence of multiple comorbidities and underlying diseases, and its incidence among hospitalized patients continues to rise.^[^ ^1^, ^2^ ^]^ Sepsis is also a disease that requires prompt diagnosis and standardized treatment,^[^ ^3^, ^4^ ^]^ If treated unproperly the mortality rate of sepsis can exceed 30–35%.^[^ ^5^ ^]^ The Surviving Sepsis Campaign: International Guidelines for Management of Sepsis and Septic Shock recommends the use of antimicrobial treatment within the first hour of onset, however, early diagnosis of sepsis remains challenging due to the complexity of the clinical context and the heterogeneity of the septic population.^[^ ^6^, ^7^, ^8^, ^9^, ^10^, ^11^, ^12^, ^13^ ^]^ Furthermore, current drugs for sepsis primarily target excessive inflammatory responses and immunosuppression and most drugs are still in the preclinical stage or exhibit limited therapeutic efficacy in clinical trials.^[^ ^14^ ^]^

Machine learning algorithms have emerged as a new technique for analyzing enormous amounts of data in the field of medicine.^[^ ^15^, ^16^ ^]^ RNA‐seq provides a comprehensive view of entire transcriptomes and has become a routine tool in biomedical research, whereas scRNA‐seq allows for the precise analysis of gene expression patterns in individual cells and the classification of cell types into specific subtypes.^[^ ^17^, ^18^, ^19^ ^]^ Previous studies employed RNA‐seq to screen four lysosome‐related genes as novel research targets for sepsis.^[^ ^20^ ^]^ Another RNA‐seq study constructed a microRNA (miRNA)‐mRNA‐protein‐protein interaction (PPI) network and identified 6 hub genes negatively correlated with the relevant miRNAs for predicting the prognosis of sepsis patients.^[^ ^21^ ^]^ Moreover, a study applying joint RNA and single‐cell sequencing methods discovered 2 mitochondrial genes (*COX7B* and *NDUFA4*) for sepsis.^[^ ^22^ ^]^ These findings encouraged us to further discover effective biomarkers for the diagnosis and treatment of sepsis applying bulk RNA‐seq and scRNA‐seq techniques based on relevant datasets obtained from public databases.

## 2 Results

### 2.1 Single‐Cell Landscape in Sepsis

**Figure 1:**
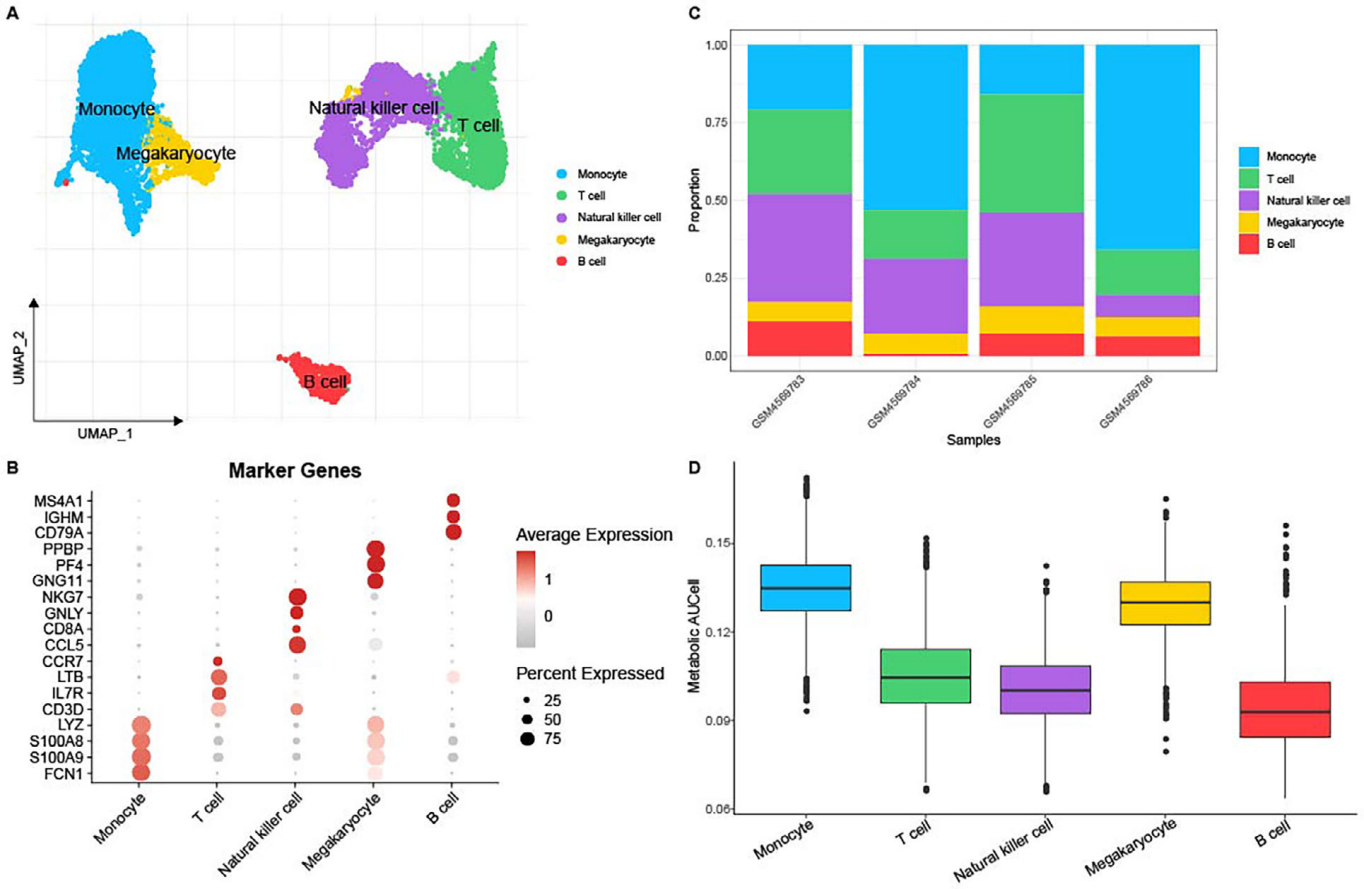
Single‐cell landscape in sepsis. A) UMAP plot on cell subpopulations based on the dataset GSE151263. B) Expression levels of relevant markers in the cell subpopulations. Each dot represents the expression of a gene, the size of the dot indicates the percentage of gene expression in the cell, and the color shade represents the average expression level of the gene. A Student's *t*‐test was used to compare differences in gene expression between subpopulations. C) The proportion of cell subpopulations in the 4 samples (GSM4569783, GSM4569784, GSM4569785, and GSM4569786). D) The AUCell enrichment score of metabolism‐related genes in each cell subpopulation.

The dataset GSE151263 containing four sepsis samples was used for the scRNA‐seq analysis. Following the procedures of data filtering, standardization, dimensionality reduction, and clustering analysis, a total of 13 382 cells were retained and grouped into five main subpopulations (**Figure** **1**A). Based on the annotation data provided, five main subpopulations were annotated, including monocytes, T cells, natural killer cells, megakaryocytes, and B cells (Figure 1B). The proportion of these subpopulations in the samples was quantified and shown in Figure 1C. The AUCell enrichment score of metabolism‐related genes in each subpopulation was also calculated, with monocytes showing the highest enrichment score (Figure 1D).

### 2.2 Heterogeneity of Monocytes in Sepsis

**Figure 2:**
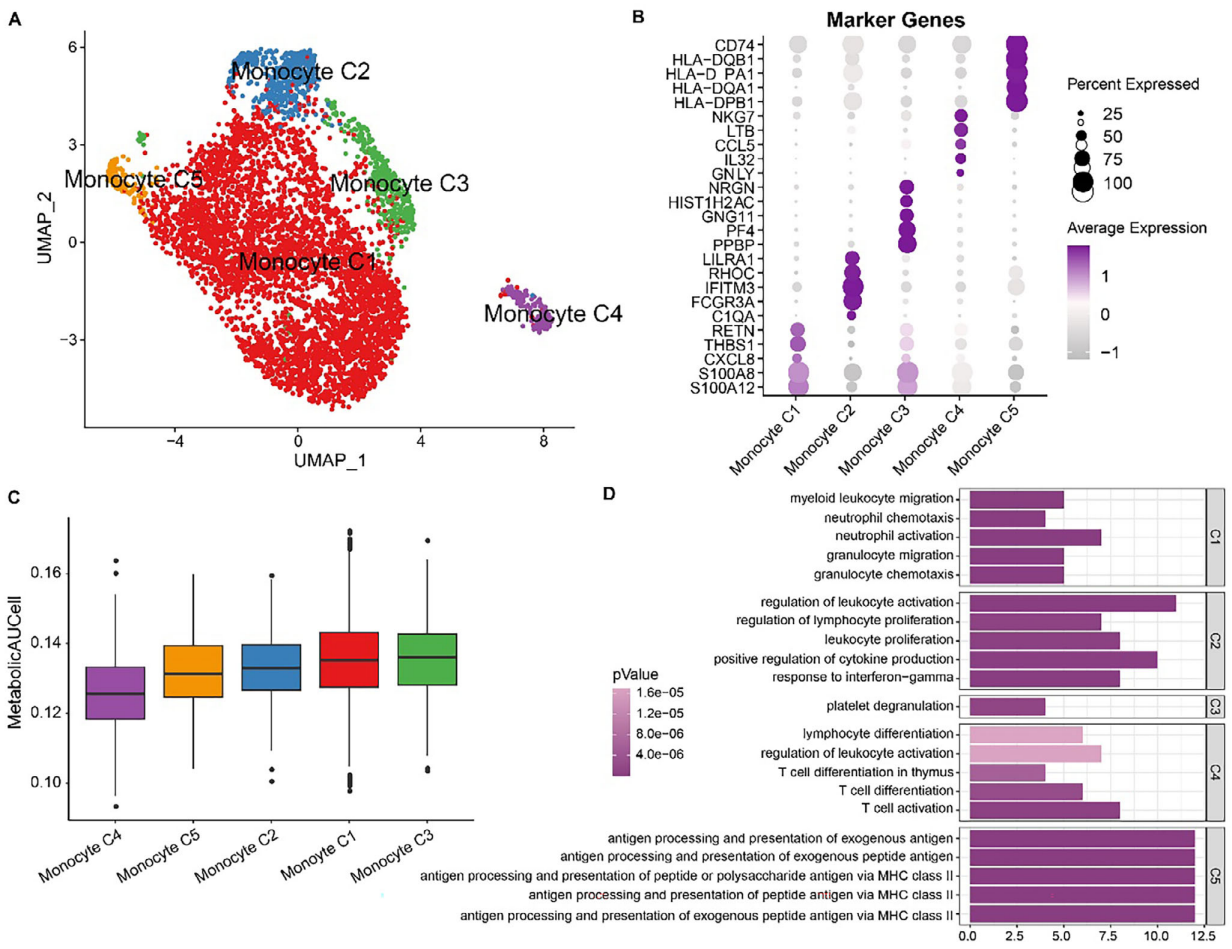
Heterogeneity of monocytes in sepsis. A) UMAP plot on the subclusters of monocytes (C1–C5). B) Genes highly expressed specific to these subclusters of monocytes (C1–C5). C) AUCell enrichment analysis of metabolism‐relevant genes in these subclusters of monocytes (C1–C5). D) GO‐BP enrichment analysis on the genes highly expressed specific to these subclusters of monocytes (C1–C5).

Dimensionality reduction and clustering were performed to group monocytes into five subclusters (C1–C5), as shown in **Figure** **2**A. Subsequently, “FindAllMarkers” was used to identify genes specifically high‐expressed in the five monocyte subpopulations (Figure 2B). Further AUCell enrichment analysis revealed the lowest score of monocyte subcluster C4 (Figure 2C). GO‐BP functional enrichment analysis on high‐expressed genes specific to these monocyte subclusters was conducted, and it was observed that these genes were mainly enriched in antigen processing and presentation of exogenous antigen, lymphocyte differentiation, and leukocyte activation (Figure 2D).

### 2.3 Cell–Cell Communication Analysis

**Figure 3:**
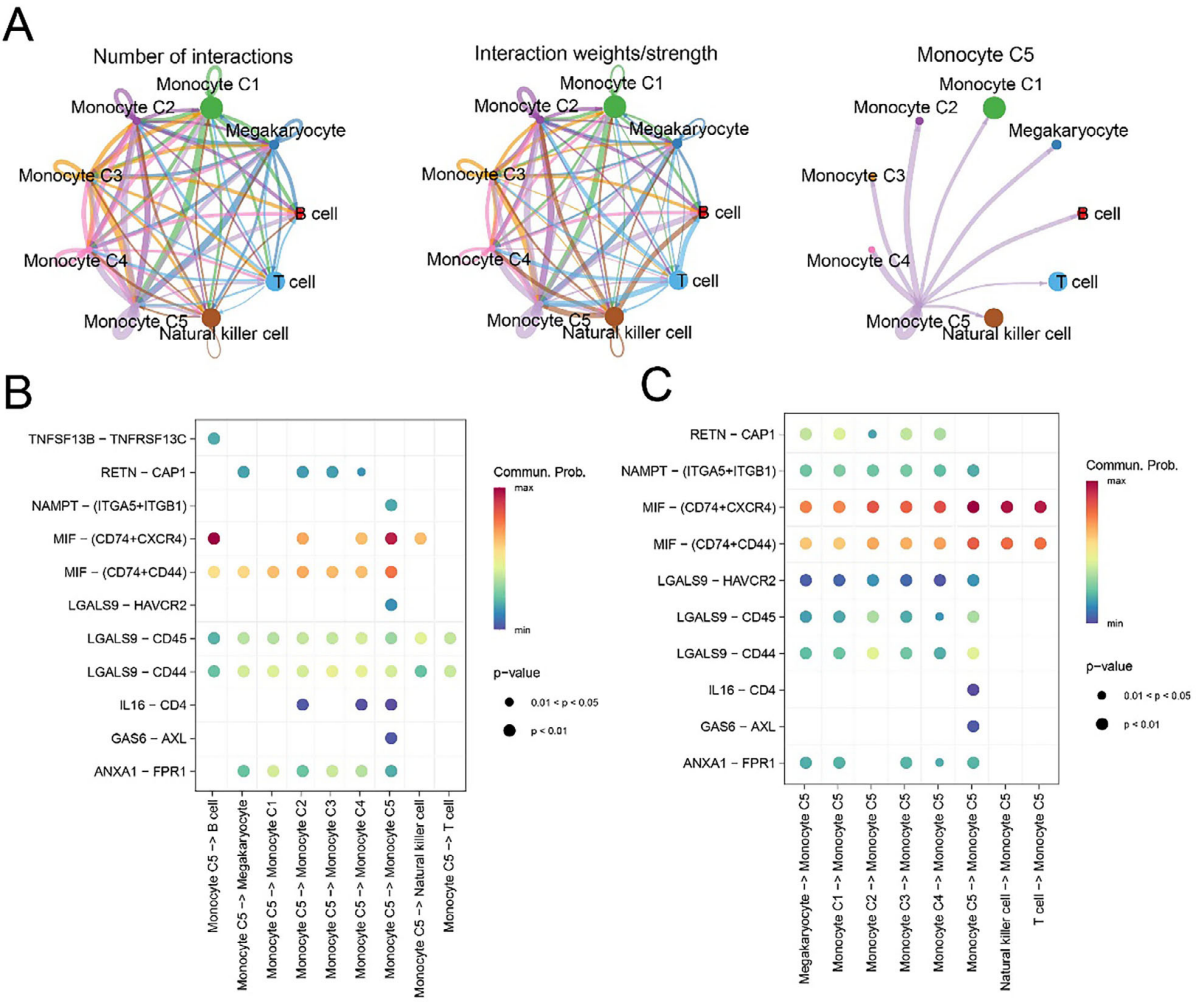
Cell–cell communication analysis. A) Cell–cell communication network of the monocyte subclusters (C1–C5). B) Ligand‐receptor pairs through which monocyte subcluster C5 could affect other subclusters of monocytes. C) Ligand‐receptor pairs through which other subclusters could affect monocyte subcluster C5 with the communication probability. Spearman's coefficient was used to evaluate the correlation between ligand‐receptor pairs.

Cell–cell communication analysis on these subclusters of monocytes (C1–C5) revealed a relatively stronger communication potential of monocyte subcluster C5 (**Figure** **3**A). Next, the data of relevant ligand‐receptor pairs in these subclusters were retrieved and analyzed, and we observed that monocyte subcluster C5 affected other subclusters through the ligand‐receptor pairs galectin 9 (LGALS9)‐CD45 and LGALS9‐CD44 (Figure 3B), while other subclusters affected monocyte subcluster C5 through ligand‐receptor pairs through MIF‐(CD74+C‐X‐C chemokine receptor type 4 (CXCR4)) and MIF‐(CD74+CD44) (Figure 3C).

### 2.4 Genes in Monocyte Subcluster C5 Identified by WGCNA

**Figure 4:**
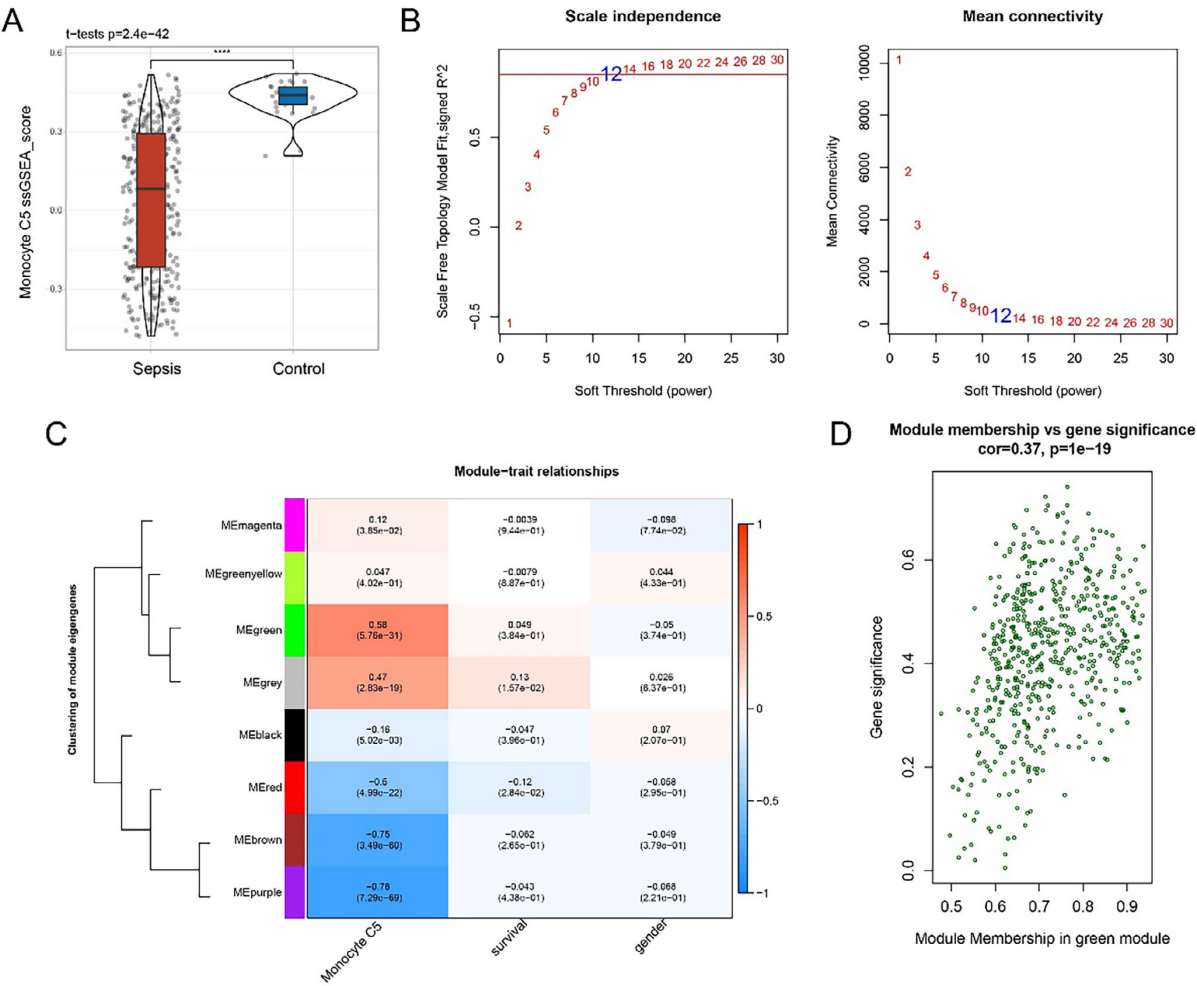
WGCNA‐based sorting on monocyte subcluster C5‐related genes. A) ssGSEA score of monocyte subcluster C5 in the sepsis and control samples based on the dataset GSE236713. Student's *t*‐test was used to compare the difference between sepsis and control groups, ^****^ *p* < 0.0001. B) Plots on determining the optimal soft threshold (β) for WGCNA based on the scale independence and mean connectivity. C) Module‐trait relationship heatmap, with shape and modules as the X and Y axis, respectively. The number in the box represents the correlation coefficient and the number in the bracket is the *p*‐value for statistical significance. D) Gene significance‐module membership plot of the MEgreen module. The correlation was calculated by Spearman's correlation coefficient with a significance *p*‐value of 1e–19 and a correlation coefficient of 0.37, indicating a strong correlation between the gene and the module members.

High‐expressed genes specific to monocyte subcluster C5 served as the background gene set. After calculating the scores of monocyte subcluster C5 in sepsis and control samples applying the single‐sample gene set enrichment analysis (ssGSEA) method, it was found that the score of subcluster C5 was evidently lower in the sepsis sample than in the control sample (**Figure** **4**A and *P* = 2.4e–42).

Gene modules related to subcluster C5 in the sepsis samples of the GSE236713 dataset were classified and subjected to the weighted gene co‐expression neywork analysis (WGCNA) under the soft threshold of 12, which was determined by the “pickSoftThreshold” function (Figure 4B). Based on the parameter minModuleSize = 80, eight specific gene modules were obtained and the module‐traits relationship heatmaps were plotted according to the results from ssGSEA (Figure 4C). It was found the MEgreen module containing a total of 563 genes was a key gene module. As shown in Figure 4D, the MEgreen module was significantly and positively correlated with the properties of subcluster C5. For this reason, we included 106 genes with gene significance (GS) ≥ 0.4 and |module membership (MM)| ≥ 0.8 as the module genes closely correlated with Monocyte C5.

### 2.5 Screening Signature Genes Specific to Subcluster C5

**Figure 5:**
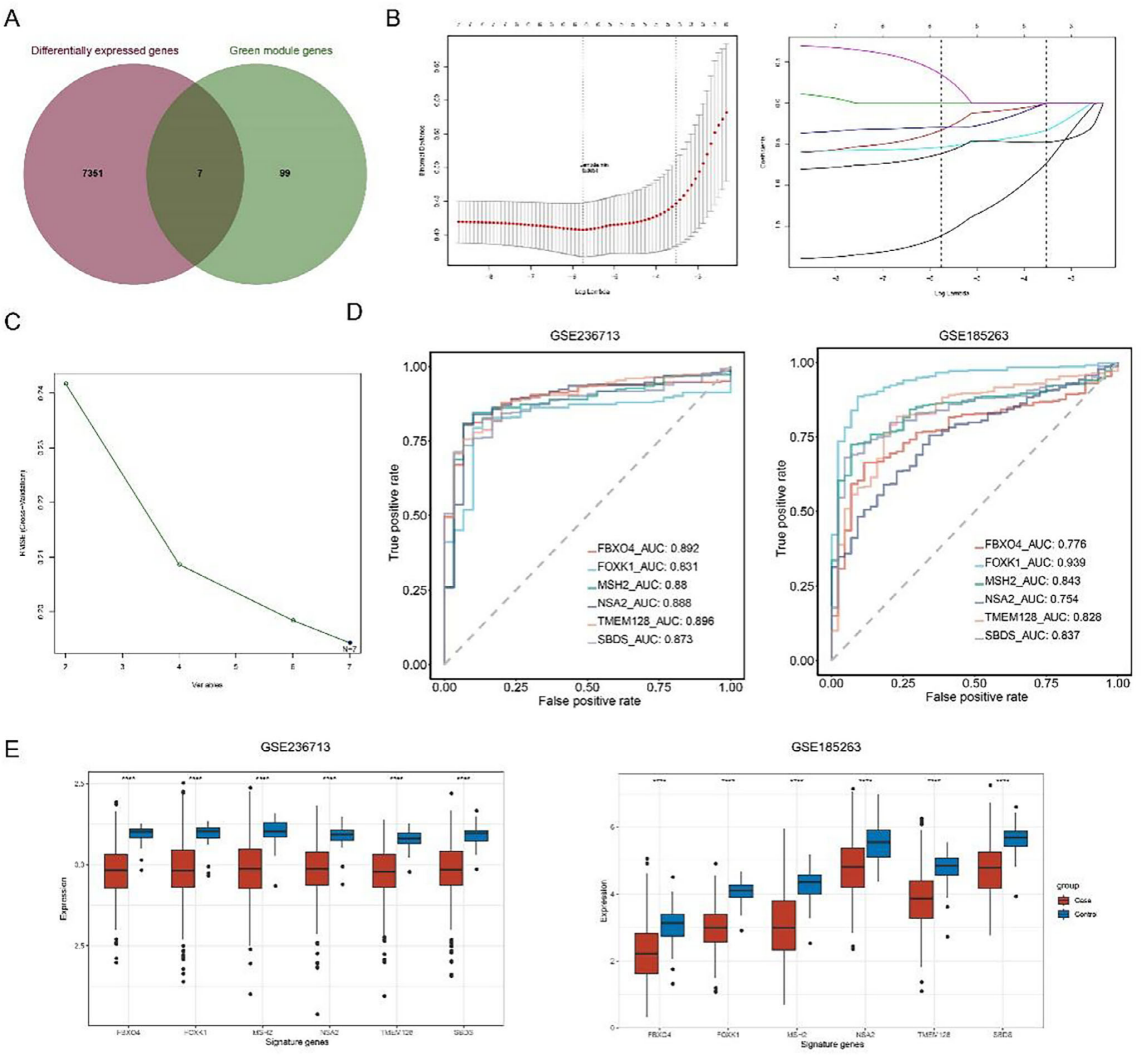
Sorting on signature genes specific to monocyte subcluster C5. A) 106 genes from the gene module MEgreen of WGCNA were intersected with the DEGs identified via the “limma” package. B) LASSO regression analysis on the 7 common genes. C) Relationship between the number of variables and root mean square error from RFE algorithm. D) Diagnostic efficacy of the 6 signature genes in the training set GSE236713 and the testing set GSE185263. E) Expression levels of the 6 signature genes in sepsis and control samples from the training set GSE236713 and the testing set GSE185263. Student's *t*‐test was used to compare the difference between sepsis and control groups, ^****^ means *p*‐value was lower than 0.0001.

The 106 genes in the MEgreen gene module intersected with the DEGs (1355 downregulated genes and 6003 upregulated genes) identified by the “limma” package. A total of seven common genes were obtained (**Figure** **5**A). LASSO regression analysis and recursive feature elimination (RFE) algorithm were applied to these seven genes. In detail, based on LASSO regression analysis, six genes were obtained when lambda.min = 0.0031 (Figure 5B). For the RFE algorithm on these seven genes, the least error was shown when the number of features was seven (Figure 5C). Then, by intersecting the results from both the LASSO regression analysis and RFE algorithm, we obtained six common genes as the key signature genes for sepsis, including F‐Box Protein 4 (*FBXO4*), Forkhead Box K1 (*FOXK1*), MutS Homolog 2 (*MSH2*), Nop‐7‐associated 2 (*NSA2*), Transmembrane Protein 128 (*TMEM128*), and *SBDS*. The diagnostic efficacy of these six genes in the training set (GSE236713) and the testing set (GSE185263) was reflected in the ROC curve. As shown in Figure 5D, the AUC values for *FBXO4, FOXK1, MSH2, NSA2, TMEM128*, and *SBDS* in the GSE236713 dataset were 0.892, 0.831, 0.88, 0.888, 0.896, and 0.873, respectively. Consistently, the AUC values for *FBXO4, FOXK1, MSH2, NSA2, TMEM128*, and *SBDS* in the GSE185263 dataset were 0.776, 0.939, 0.843, 0.754, 0.828, and 0.837, respectively. Furthermore, compared to the control samples, we found that the expressions of all these six signature genes were lower in sepsis samples in both training sets GSE236713 and GSE185263 (Figure 5E).

### 2.6 Immune Cell Infiltration Analysis and Correlation Analysis on the Biomarkers

**Figure 6:**
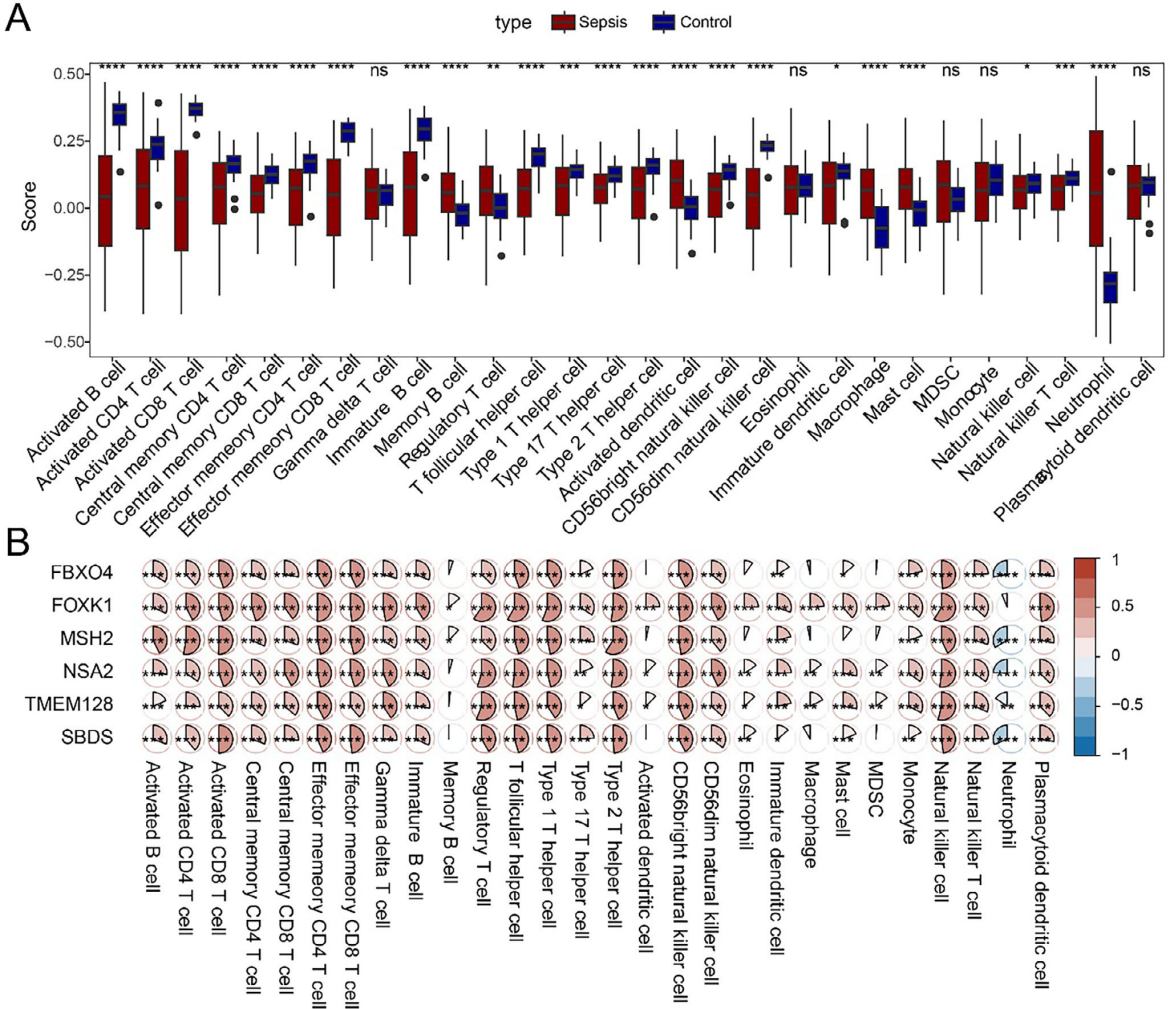
Immune cell infiltration analysis and correlation analysis with the relevant biomarkers. A) Difference in immune cell infiltration status based on different samples. B) Correlation analysis on the immune cell infiltration and relevant biomarkers in sepsis samples. In the figures, ^*^ means the *p*‐value was lower than 0.05, ^**^ means the *p*‐value was lower than 0.01, ^***^ means the *p*‐value was lower than 0.001, and ^****^ means the *p*‐value was lower than 0.0001) and data without statistical significance (*p*‐value > 0.05) were labeled with “ns” (abbreviation for no significance).

The degree of immune infiltration of 23 types of immune cells in sepsis and control samples was compared using ssGSEA (**Figure** **6**A), the results of which showed that most of the immune cells had a lower degree of infiltration. Spearman correlation analysis on the infiltration score and the six signature genes revealed that these genes were positively correlated with most immune cells but negatively correlated with neutrophils (Figure 6B).

## 3 Discussion

Previous studies utilized both RNA‐seq and scRNA‐seq data to develop a novel prognostic model and analyze drug efficacy in sepsis, aiming to provide novel insights into the diagnosis of sepsis.^[^ ^23^ ^]^ Similarly, the present study also employed both RNA‐seq and scRNA‐seq analyses to discover relevant biomarkers for sepsis. Our scRNA‐seq analysis revealed a relatively higher percentage of monocytes. Subsequent investigation into the heterogeneity of monocytes grouped five main subclusters (C1–C5). Cell–cell communication analysis revealed a significant role of subcluster C5 in influencing other subclusters of sepsis. After performing WGCNA on the subcluster C5, we identified six specific genes as the feature genes. Additionally, most immune cells showed lower infiltration in sepsis, and these six genes exhibited a positive correlation with most immune cells but a negative correlation with neutrophils. This evidence further supported the critical role of monocytes in sepsis, providing novel insights for the diagnosis and treatment of sepsis in clinical practice.

Based on the results from cell–cell communication analysis on the five main subclusters of monocytes, we identified potential ligand‐receptor pairs through which monocyte subcluster C5 and other subclusters affected each other. Studies have extensively explored the potential involvement of monocytes in sepsis with scRNA‐seq. Based on the study of Yao et al., human leukocyte antigen DR (HLA‐DR)^low^S100A^high^ monocytes with immunosuppressive effects were found to be prominently enriched in septic patients.^[^ ^24^ ^]^ Another study revealed an increase in CAP‐1+ monocytes in sepsis, concurrent with a validated IL16‐CD4 ligand‐receptor pair and enhanced IL16 signaling.^[^ ^25^ ^]^ In this study, after confirming the heterogeneity of monocytes in sepsis, it was observed that monocyte subcluster C5 had a relatively stronger communication potential, and that monocyte subcluster C5 affected other subclusters via the common ligand‐receptor pairs LGALS9‐CD45 and LGALS9‐CD44. *LGALS9* is a member of the family of galectin, which is widely expressed and involved in immune regulation.^[^ ^26^ ^]^ Moreover, inhibition of *LGALS9* could result in the activation and differentiation of monocytes in sepsis patients.^[^ ^27^ ^]^ This evidence pointed to a potential role of *LGALS9* in monocyte subcluster C5 and suggested that these ligand‐receptor pairs may be implicated in sepsis, while aberrant *LGALS9* expression within monocytes may be a potential biomarker for diagnosing sepsis.

Subsequently, WGCNA was applied to determine high‐expressed biomarkers specific to monocyte subcluster C5 as the background gene set. Based on the MEgreen gene modules, a total of 106 genes were considered as the high‐expressed genes specific to the C5 monocyte subpopulation. To reduce the number of these genes for our current analysis, these 106 genes were intersected with the DEGs to obtain seven common genes. After subjecting the seven genes to LASSO regression analysis and RFE algorithm, six signature genes were finally determined, including *FBXO4, FOXK1, MSH2, NSA2, TMEM128*, and *SBDS*. *FBXO4*, alternatively known as FBX4, is a member of the F‐box protein family that contains a conserved F‐box domain and fulfills its biological functions via forming the FBOX4‐S‐phase kinase‐associated protein 1 (SKP1) and FBOX4‐Cullin1 complexes.^[^ ^28^ ^]^ Forkhead box (FOX) proteins comprise a group of evolutionarily conserved transcription factors that are characterized by a specific DNA‐binding domain named the forkhead/winged helix domain.^[^ ^29^ ^]^ Research indicated that the members of this family play critical roles in various processes during embryonic development, including lineage fate determination, cell cycle dynamics, aging, metabolic processes, regulation of stem cells, and chromatin remodeling, therefore serving as pioneer factors.^[^ ^30^, ^31^ ^]^ FOXK1, a member of the Foxk family, has been shown to regulate the expressions of signal transducer and activator of transcription (STAT)1 and STAT2, which all play an important role in regulating macrophage antiviral immunity.^[^ ^32^ ^]^ MSH2 can interact with AT‐rich interaction domain 1A (*ARID1A*) and potentiate therapeutic immunity unleashed via immune checkpoint blockade.^[^ ^33^ ^]^ NSA2 has been identified as a nucleolus protein capable of modulating both cell proliferation and cell cycle.^[^ ^34^ ^]^ *TMEM128* is a protein‐coding gene belonging to those proteins that have been predicted to be an integral component of the membrane (provided by Alliance of Genome Resources, Apr 2022). The *SBDS* gene encodes a highly conserved protein critical for ribosome biogenesis, while loss of *SBDS* can suppress cell growth.^[^ ^35^, ^36^ ^]^ Our current study applied a series of computational analyses to reveal the involvement of these genes in sepsis, as evidenced by the discovery of their diagnostic efficacy and their expression profile in sepsis patients. Further immune infiltration analysis showed that these six genes were positively correlated with most immune cells but negatively correlated with neutrophils. Nonetheless, the specific involvement of these signature genes in sepsis still requires further laboratory or experimental analyses, which was also a limitation of this study.

## 4 Conclusion

Based on the results from both RNA‐seq and scRNA‐seq analyses of the current study, we revealed a specific cell–cell communication pattern and heterogeneity of the monocytes in sepsis patients. In detail, monocyte subcluster C5 was confirmed to affect other monocyte subclusters through the ligand‐receptor pairs LGALS9‐CD45 and LGALS9‐CD44. Further machine learning algorithms based on this particular subcluster identified six signature genes for future laboratory or experimental analyses, providing novel targets for the diagnosis and treatment of sepsis.

## 5 Experimental Section

### Data Source and Pre‐Processing

The datasets GSE151263 for scRNA‐seq analysis, GSE236713 (containing 324 sepsis samples and 30 normal samples), and GSE185263 (containing 348 sepsis samples and 44 normal samples) for RNA‐seq analysis were all downloaded from Gene Expression Omnibus (GEO, https://www.ncbi.nlm.nih.gov/geo/). Meanwhile, a list of 2752 genes encoding metabolic enzymes and transporters was extracted from a published study.^[^ ^37^ ^]^

For data pre‐processing, first, the dataset GSE151263 was filtered based on the criteria that each gene was expressed in at least 3 cells and each cell expressed over 200 genes. Qualified cells with a mitochondrial gene proportion < 15%, gene number between 500 and 6000, unique molecular identifier (UMI) > 200, and log10GeneperUMI > 0.8 were retained for subsequent analysis. After data standardization, highly variable genes were identified and gene expressions were scaled using the “ScaleData” function. The “RunPCA” function was used for principal component analysis (PCA) and the “harmony” R package was adopted to remove the batch effects.^[^ ^38^ ^]^ The first 30 PCs were subjected to dimensionality reduction using uniform manifold approximation and projection. Cell subpopulations were then classified employing the functions “FindNeighbors” and “FindClusters” and annotated based on the specific marker genes of the CellMarker2.0 database.^[^ ^39^, ^40^ ^]^ The results before and after the quality control are shown in Figure S1 (Supporting Information).

For the dataset GSE236713, the annotation file in the chip platform was downloaded and the probes were matched to genes based on the relevant annotation file. Maximized gene expression was taken when multiple probes matched to only one gene. For the GSE185263 dataset, the count matrix was converted to TPM format and log2‐transformed. Gene symbol IDs were subsequently derived from Ensembl gene IDs. Finally, genes exhibiting multiple gene symbol IDs were normalized to the median value.

### ssGSEA

The ssGSEA could calculate the enrichment score to indicate the absolute enrichment of a gene set in the sample within a specified dataset.^[^ ^41^ ^]^ In this study, the relative enrichment of a gene set in each sample was estimated by comparing its gene expression data to the specified gene set. Each ssGSEA enrichment score represents the extent to which a specific gene set was up or down‐regulated in a sample.

### Identification of Differentially Expressed Genes (DEGs)

DEGs of cell subpopulations were identified using the “limma” R package under the thresholds of adjusted.*p*.value<0.05 and |log2FC|> log(2).^[^ ^42^, ^43^, ^44^ ^]^

### Functional Enrichment Analysis

The genes specifically higher‐expressed in the subclusters of monocytes were subjected to the functional enrichment analysis of Gene Ontology in biological processes (GO‐BPs) terms using the “ClusterProfiler” package.^[^ ^45^ ^]^

### WGCNA

WGCNA was a systems biology method used to characterize gene correlation between different samples and could be used to identify highly synergistically variable gene sets. The method identified candidate biomarker genes or therapeutic targets based on the endlinkage of gene sets and the association between gene sets and phenotypes.^[^ ^46^ ^]^ Here, a sum of 324 sepsis samples from the GSE236713 dataset were subjected to WGCNA analysis. First, a sample clustering tree was created using unsupervised cluster analysis to identify and remove outliers. After this, the correlation coefficient among the genes was calculated by Pearson analysis to construct a similarity matrix. In order to ensure a scale‐free network, the soft threshold (β) was determined using the “pickSoftThreshold” function. Next, the adjacency matrix converted from the expression matrix was transformed into a topological matrix. Based on the topological overlap matrix, the relevant genes were clustered via the average linkage hierarchical clustering method. After classifying gene modules with the dynamic shear method, the eigengenes in specific gene modules were calculated and the clustering analysis was performed on the modules to combine close modules into a new module with the following parameters: height = 0.25, deepSplit = 2, and minModuleSize = 80. Next, relevant module‐specific genes were screened according to GS ≥ 0.4 and |MM| ≥ 0.8.

### Identification of Feature Genes in the Specific Cell Subclusters

The relevant genes in the gene modules from WGCNA were intersected with the DEGs, and common genes were subjected to LASSO regression analysis using the “glmnet” package^[^ ^47^ ^]^ and RFE algorithm using the “caret” package.^[^ ^48^ ^]^ The diagnostic efficacy of the common genes from these two analyses was validated in datasets GSE236713 (the training set) and GSE185263 (the testing set). The receiver operating characteristic (ROC) curves were plotted and the area under the curve (AUC) was calculated accordingly.

### Immune Infiltration Analysis

The gene set enrichment levels of immune cells were assessed using ssGSEA. The infiltration levels of 28 types of immune cells were computed utilizing the R package GSVA based on the expressions of genes in a total of 28 established gene sets.^[^ ^49^ ^]^

### Cell–Cell Communication Analysis

The “CellChat” package was applied for the analysis.^[^ ^25^, ^50^ ^]^ Specifically, the “createCellchat” function was utilized to construct relevant objects, and overexpressed ligand‐receptor pairs were identified by the functions of “identifyOverExpressedGenes” and “identifyOverExepressedInteractions.” The potential interactions between ligand‐receptor pairs were inferred using the “computeCommunProb” function, and the results were visualized by the “netVisual_bubble” function.

### Statistical Analysis

All statistical analyses were realized with R software (version 3.6.3). The Wilcoxon rank‐sum test and Student's *t*‐test were employed to compare the two groups based on the outcomes of the Shapiro–Wilk test. Correlation analysis was conducted utilizing Spearman's coefficient. The mean and standard deviation (SD) of the data were shown. Under the threshold of *p* < 0.05, the statistical significance was marked with the asterisks (^*^ means *p* < 0.05, ^**^ means *p* < 0.01, ^***^ means *p* < 0.001, and ^****^ means *p* < 0.0001), while data without statistical significance (*p* > 0.05) were labeled with “ns” (abbreviation for ‘no significance’).

### Availability of Data and Material

The public dataset used in this study is available in GSE151263 (https://www.ncbi.nlm.nih.gov/geo/query/acc.cgi?acc = GSE151263), GSE263713 (https://www.ncbi.nlm.nih.gov/geo/query/acc.cgi?acc = GSE263713) and GSE185263 (https://www.ncbi.nlm.nih.gov/geo/query/acc.cgi?acc = GSE185263).

## Conflict of Interest

The authors declare no conflict of interest.

## Author Contributions

Q.Y. and Y.D. contributed equally to this study. All authors contributed to this present work: Q.L.Y. and Y.H.D. designed the study, J.T.L. and R.T. acquired the data, and J.Y.L. and S.Z. interpreted the data. S.Z., G.Y.H., and Q.L.Y. improved the figure quality, Q.L.Y., R.T., and Y.H.D. drafted the manuscript, and Q.L.T., J.T.L., and S.Z. revised the manuscript. All authors read and approved the manuscript.

## Supporting information



Supporting Information

## Data Availability

The data that support the findings of this study are available from the corresponding author upon reasonable request.
