# Supplementary material for: An In‐Silico Study to Identify Relevant Biomarkers in Sepsis Applying Integrated Bulk RNA Sequencing and Single‐Cell RNA Sequencing Analyses
Source: Glob Chall. 2025 Mar 13;9(4):2400321. doi: 10.1002/gch2.202400321 (PMC12003214; doi:10.1002/gch2.202400321)
Supplement: Supplementary file 1 — Supporting Information [file GCH2-9-2400321-s001.docx]

**Supplementary Fig 1.** Results on data processing before and after quality control. (A) Parameters of nFeature_RNA, nCount_RNA and percent.mt based on the dataset GSE151263 containing 4 samples (GSM4569783, GSM4569784, GSM4569785 and GSM4569786). (B) Gene counts in 4 samples (GSM4569783, GSM4569784, GSM4569785 and GSM4569786). (C) UMAP plot based on the 4 samples (GSM4569783, GSM4569784, GSM4569785 and GSM4569786).

**
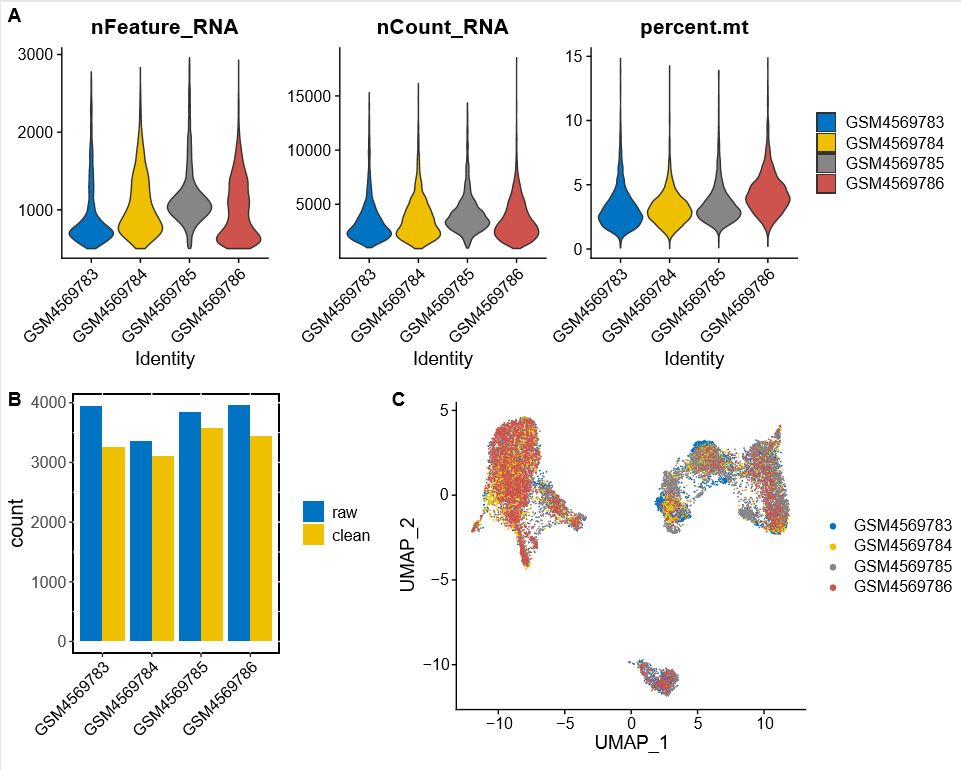
**
